# Intrinsic and synaptic properties of hippocampal CA1 pyramidal neurons of the Wistar Audiogenic Rat (WAR) strain, a genetic model of epilepsy

**DOI:** 10.1038/s41598-018-28725-y

**Published:** 2018-07-10

**Authors:** Alexandra Olimpio Siqueira Cunha, Cesar Celis Ceballos, Júnia Lara de Deus, Rodrigo Felipe de Oliveira Pena, José Antonio Cortes de Oliveira, Antonio Carlos Roque, Norberto Garcia-Cairasco, Ricardo Maurício Leão

**Affiliations:** 1 Department of Physiology, School of Medicine of Ribeirão Preto, University of São Paulo, Ribeirão Preto, SP Brazil; 2 Department of Physics, School of Philosophy, Sciences and Letters of Ribeirão Preto, University of São Paulo, Ribeirão Preto, SP Brazil

## Abstract

Despite the many studies focusing on epilepsy, a lot of the basic mechanisms underlying seizure susceptibility are mainly unclear. Here, we studied cellular electrical excitability, as well as excitatory and inhibitory synaptic neurotransmission of CA1 pyramidal neurons from the dorsal hippocampus of a genetic model of epilepsy, the Wistar Audiogenic Rat (WARs) in which limbic seizures appear after repeated audiogenic stimulation. We examined intrinsic properties of neurons, as well as EPSCs evoked by Schaffer-collateral stimulation in slices from WARs and Wistar parental strain. We also analyzed spontaneous IPSCs and quantal miniature inhibitory events. Our data show that even in the absence of previous seizures, GABAergic neurotransmission is reduced in the dorsal hippocampus of WARs. We observed a decrease in the frequency of IPSCs and mIPSCs. Moreover, mIPSCs of WARs had faster rise times, indicating that they probably arise from more proximal synapses. Finally, intrinsic membrane properties, firing and excitatory neurotransmission mediated by both NMDA and non-NMDA receptors are similar to the parental strain. Since GABAergic inhibition towards CA1 pyramidal neurons is reduced in WARs, the inhibitory network could be ineffective to prevent the seizure-dependent spread of hyperexcitation. These functional changes could make these animals more susceptible to the limbic seizures observed during the audiogenic kindling.

## Introduction

Epilepsy is a set of neurological disorders that has as a common symptom the appearance of sudden events of hypersynchronization and hyperactivity of neurons^1^. Despite the great amount of scientific literature on epilepsy, the basic mechanisms of seizures onset and spread still remain unclear. Thus, animal models offer ways to test hypothesis on those mechanisms, and can help to identify novel targets and consequently more efficient tools^2^.

Acute audiogenic seizures are generalized reflex tonic-clonic seizures induced by a high intensity sound (e.g. 120 dB), regardless of its frequency. Although they are rare in humans, audiogenic seizures are very well characterized in several strains of rodents, with stereotyped behaviors and epileptiform electroencephalographic activity initially restricted to the auditory brainstem^3–5^. Nonetheless, upon repetition of the sound, the animals start to exhibit behavioral patterns typical of limbic seizures, with epileptiform discharges spreading through amygdala, hippocampus and auditory cortex, a phenomenon known as limbic recruitment^5–8^.

Auditory stimulation impacts the hippocampus in complex ways. There are evidences, for example, that hippocampal place cells respond to an auditory dimension task^9^. Also, we showed that repeated high intensity sound stimulation inhibits hippocampal long-term potentiation (LTP)^10,11^.

According to our previous findings, naïve Wistar Audiogenic Rats (WARs) showed memory impairments in spatial performance in the Morris Water Maze and a slower development of LTP^10^. Additionally, a decreased GABAergic inhibition was observed in dissociated neurons from the hippocampus of newborn WARs^12^ as well as field potentials^13^ recorded in hippocampal slices of adult WARs. Finally, a recent study showed, in addition to other morphological alterations, marked decrease in the volume in the CA3 of adult WARs^14^. In the current study we characterized membrane electrical properties of CA1 pyramidal neurons from the dorsal hippocampus of naive WARs, as well as synaptic neurotransmission, to verify possible alterations that could be relevant for the seizure-dependent spread of hyperexcitation when challenged by acute or chronic audiogenic seizures.

## Results

### CA1 pyramidal cells of WARs are more hyperpolarized than those from Wistar cells

**Figure 1:**
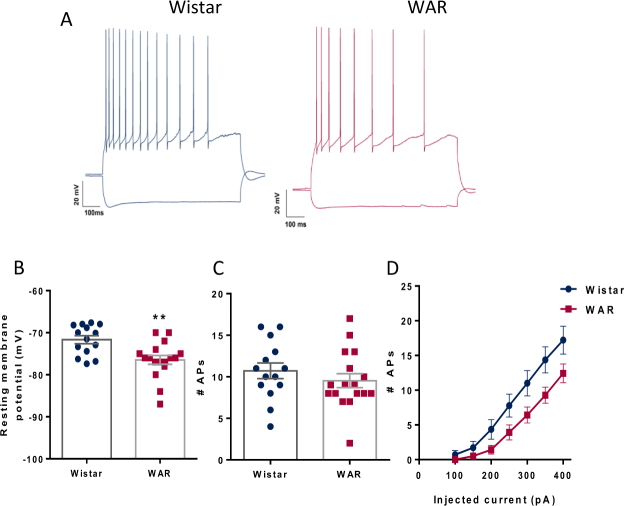
Electrical properties and firing in pyramidal neurons. (**A**) Voltage changes in response to current injections for Wistar (blue) and WAR (fucsia). (**B**) Resting membrane potential. (**C**) Number of action potentials 100 pA over rheobase and (**D**). FI curves. N = 14 cells from 6 Wistars and N = 17 cells from 8 WARs **P < 0.01.

CA1 pyramidal neurons from WARs (n = 17) and Wistar rats (n = 14) had similar firing patterns (Fig. 1A) typical to what has been previously reported for CA1 pyramidal neurons (Wheeler *et al*., 2015). The resting membrane potential of WAR neurons are more hyperpolarized than Wistar neurons (Wistar = −72 ± 0.9 mV; WAR = −77 ± 1.04 mV; P = 0.0008; Fig. 1B), but we did not observe statistical differences in input resistance, membrane time constant or depolarization sag (Table 1) between neurons from WAR and Wistar rats. The analysis of FI curves showed that WAR cells fire less action potentials than Wistar cells [F (1,26) = 4.39, p = 0.046, for main effect], although at a fixed value of 100 pA over rheobase, we did not see statistical differences between the two groups (Wistar = 10.7 ± 0.95 Hz; WAR = 9.14 ± 0.89 Hz; P = 0.23; Fig. 1C,D), suggesting that this difference was caused by the more hyperpolarized resting membrane potential of WAR neurons. Also, action potential parameters did not differ between groups (Table 1).

**Table 1:** Intrinsic Membrane properties and action potential kinetic parameters of CA1 pyramidal neurons of WAR and Wistar animals.

Intrinsic Membrane Properties and Action Potential Parameters			
	Wistar	WAR	p
R_input_ (MΩ)	69 ± 3	64 ± 3	0.27
τ (ms)	16.8 ± 1.2	13.7 ± 1	0.06
Sag (mV)	3.1 ± 0.5	3.1 ± 0.3	0.99
AP_amplitude_ (mV)	104.5 ± 1.6	105.6 ± 1.3	0.61
Half width (ms)	0.97 ± 0.02	0.9 ± 0.1	0.91
Fast_AHP_ (mV)	−69.4 ± 0.9	−70.6 ± 1.5	0.52
AP_threshold_ (mV)	−61.9 ± 0.7	−60.4 ± 0.9	0.24

Data are represented as mean ± SEM. Unpaired t-test with p < 0.05 considered as significant.

### Glutamatergic excitatory neurotransmission is similar in WAR and Wistar pyramidal neurons

**Figure 2:**
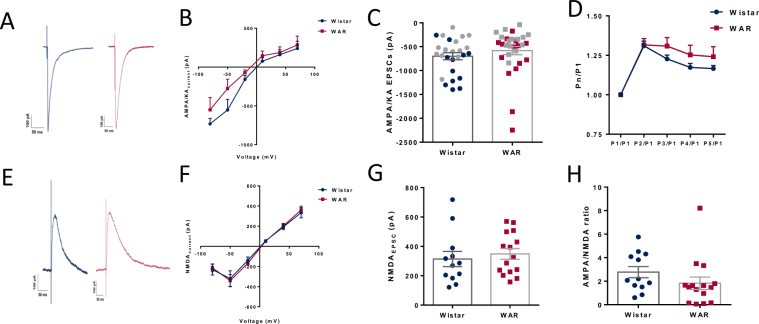
Glutamatergic neurotransmission. (**A**) Representative traces of evoked EPSCs in cells from Wistar (blue) and WAR (fucsia) at −70 mV. (**B**) AMPA/KA peak currents at different voltages. (**C**) Mean peak currents at −70 mV recorded with CsCl (grey symbols) and KGlu as main ion in the internal solution. (**D**) Trains of 5 evoked pulses at −70 mV show facilitation. (**E**) Representative traces of NMDA peak currents evoked at +80 mV in the presence of DNQX (10 µM). (**F**) NMDA peak currents at different voltages. (**G**) Mean peak currents at +80 mV. H. NMDA/AMPA ratio obtained by dividing the peak current at +80 mV (with DNQX 10 µM) by the peak current at −70 mV. N = 12 cells from 9 Wistars and N = 15 from 7 WARs. In C, N = 26 cells from 18 Wistars and N = 30 cells from 14 WARs.

In Fig. 2 we show EPSCs mediated by AMPA/KA and NMDA receptors. Our data show that AMPA/KA-mediated EPSCs from both strains were similar in all tested voltages (Fig. 2B, n = 12 for Wistar and n = 15 for WARs). The amplitudes of EPSCs at −70 mV are similar in cells from Wistar (n = 14) and WARs (n = 15) (Wistar = −523.8 ± 71.07 pA; WAR = −388.3 ± 56.6 pA; P = 0.13; Fig. 2C), as well as the rise times (Wistar = 5.54 ± 0.6 ms; WAR = 5.36 ± 0.24 ms; P = 0.77), decay times (Wistar = 44.4 ± 4.13 ms; WAR = 46.7 ± 3.7 ms; P = 0.68) and half-widths (Wistar = 14.39 ± 1.3 ms; WAR = 12.9 ± 0.72 ms; P = 0.32). The AMPA/KA conductances were similar between the two groups (Wistar = 14.39 ± 1.3 ms, n = 12; WAR = 12.9 ± 0.72 ms, n = 15; P = 0.32). We also found that synapses from both animals presented similar short-term facilitation of AMPA/KA currents [F (1, 29) = 1.063; P = 0.3112, for main effect, n = 14 for Wistar and n = 15 for WARs] (Fig. 2D).

NMDA EPSCs had similar amplitudes in all tested voltage steps in the two groups of neurons (Fig. 2F, n = 12 for Wistar and n = 15 for WARs). NMDA at +80 mV EPSCs from CA1 neurons from Wistar and WARs had similar amplitudes (I_NMDA@+80 mV_: Wistar = 313.9 ± 51.8 pA, n = 14; WAR = 348.3 ± 36.6 pA, n = 15; P = 0.58, Fig. 2G; rise times (Wistar = 3.3 ± 0.5 ms; WAR = 3.4 ± 0.34 ms; P = 0.86), decay times (Wistar = 305.3 ± 21.5 ms; WAR = 344 ± 22.6 ms; P = 0.23) and half-widths (Wistar = 14.39 ± 1.3 ms; WAR = 12.9 ± 0.72 ms; P = 0.32). We did not observe any differences in NMDA conductances in depolarized potentials (Wistar = 4.68 ± 0.8 nS; WAR = 5.29 ± 0.48 nS; P = 0.5) The ratio between AMPA/KA and NMDA EPSCs was not significantly different (Wistar = 0.398 ± 0.075; WAR = 0.67 ± 0.11; P = 0.2; Fig. 2H).

### Neurons from WAR animals receive less GABAergic spontaneous IPSCs

**Figure 3:**
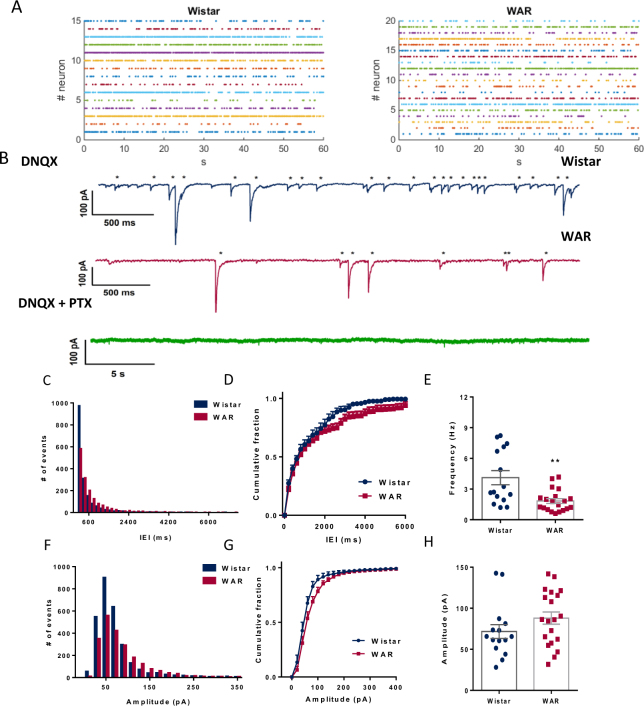
GABAergic spontaneous IPSCs. (**A**) Raster plots displaying all events in 1 min of recording from all patched cells. (**B**) Representative traces of spontaneous IPSCs with detected events marked (*) for Wistar (blue) and WAR (fucsia) cells. All events disappeared after addition of picrotoxin (20 µM) in the bath. (**C**) Histograms with the sum of all inter-event intervals (IEI; Bin width = 200 ms). (**D**) Cumulative fraction of events of IEIs. (**E**) Mean global frequency of IPSCs for each group of animals. (**F**) Histogram showing distribution of events by amplitude recorded during 1 min (Bin width = 20pA). (**G**) Cumulative fraction of amplitudes per group. (**H**) Mean amplitude of all detected IPSCs per group. **P < 0.01. N = 15 cells from 7 Wistars and N = 20 from 7 WARs.

Spontaneous GABAergic IPSCs recorded in CA1 pyramidal cells, reflect the activity of the interneuron network over principal excitatory cells. To determine whether genetic selection of the audiogenic phenotype had impacted GABAergic neurotransmission, we analyzed spontaneous IPSCs. Figure 3A shows raster plots of the sIPSCs over a 1-minute window. In Fig. 3B, in turn, we show typical IPSCs from Wistar and WAR neurons, and below (in green) the effect of applying the GABA_A_ receptor antagonist picrotoxin (20 and 100 µM), confirming the GABAergic nature of the currents.

We counted more events in total from Wistar neurons than from WAR neurons (Fig. 3C) suggesting a more active inhibitory network onto the CA1 pyramidal cell from Wistar animals. Accordingly, the analysis of IEI distributions showed significant differences between the two groups (KS test, p = 0.002. Fig. 3D) with a distribution of IEIs shifted to the right in WARs. The mean frequency of IPSCs was significantly smaller in cells from WARs as compared with Wistar cells (Wistar = 4.11 ± 0.68 Hz; WAR = 1.8 ± 0.23 Hz; P = 0. 0014; Fig. 3E). The analysis of the coefficient of variation (CVs) of the IEI was similar in both groups indicating the same irregular pattern of activity in time in the two groups (Wistar = 99.35 ± 5.24%; WAR = 107.1 ± 7.07%; P = 0.4; Fig. 3F).

The amplitudes distributions followed a skewed Gaussian pattern and were similar between the groups (Fig. 3F). We did not observe differences in amplitude cumulative distribution (KS test, p = 0.95), and in mean amplitudes (Wistar = 71.6 ± 8.4 pA; WAR = 87.9 ± 7.4 pA; p = 0.15, Fig. 3H) and the amplitude CVs were similar between the two groups, showing similar variabilities among them (Wistar = 63.29 ± 6.3%; WAR = 63.38 ± 4.3%; p = 0.99). ISPCs from both groups of animals presented similar rise times (Wistar = 1.15 ± 0.08 ms; WAR = 1.1 ± 0.05 ms; p = 0.6), decay times (τ_fast_, Wistar = 3.52 ± 0.47 ms; WAR = 2.7 ± 0.23 ms; p = 0.11 and τ_slow_, Wistar = 38.65 ± 5.7 ms; WAR = 28.78 ± 3 ms; p = 0.1) and half-widths (Wistar = 3.15 ± 0.3 ms; WAR = 2.5 ± 0.2 ms; p = 0. 12).

### Miniature GABAergic currents are faster and less frequent in WARs

**Figure 4:**
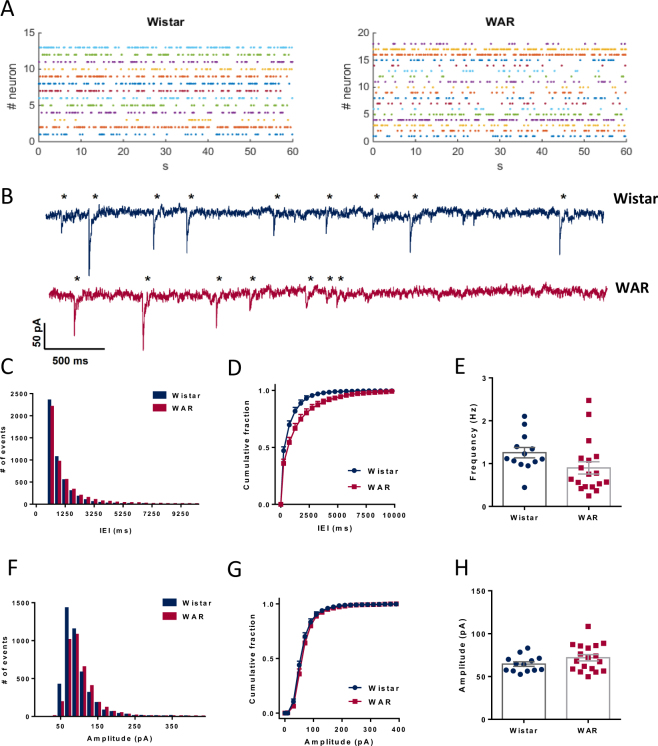
Frequency and IEI of quantal GABAergic mIPSCs. (**A**) Raster plots displaying all events in 1 min from all patched cells. (**B**) Representative traces of spontaneous mIPSCs with detected events marked (*) for Wistar (blue) and WAR (fucsia) cells. (**C**) Histogram showing distribution of events by IEI recorded for 5 minutes (Bins of 200 ms). (**D**) Cumulative fraction of IEI per group. (Bins of 50 ms). (**E**) Mean global frequency of mIPSCs. (**F**) Histogram showing distribution of events by amplitude recorded for 5 minutes (Bins of 20 pA). (**G**) Cumulative fraction of amplitudes per group. (**H**) Mean amplitudes. N = 13 cells from 7 Wistars and N = 18 from 7 WARs.

GABAergic currents were recorded in the presence of TTX. Application of TTX inhibited more the frequency of the IPSCs in neurons from Wistar rats than from cells of WAR rats (Wistar = 49% and WAR = 30.5%). In Fig. 4 we show raster plots of mIPSCs in 1-minute windows for each cell together with typical mIPSCs (A-B). After TTX, the cumulative distributions of the IEIs from WARS were again shifted to the right, with more events separated by longer IEIs (>1 second; KS test, p < 0.0001; Fig. 4C,D) and the mean frequency of WAR mIPSCs was lower than that of Wistar mIPSCs, although this difference did not reach significance (Wistar = 1.25 ± 0.12 Hz; WAR = 0.89 ± 0.14 Hz; P = 0.084; Fig. 4E). The CVs were similar indicating the same irregular pattern of mIPSCs appearance for both groups of animals (Wistar = 114.7 ± 11.7%; WAR = 106 ± 2.7%; p = 0.4).

**Figure 5:**
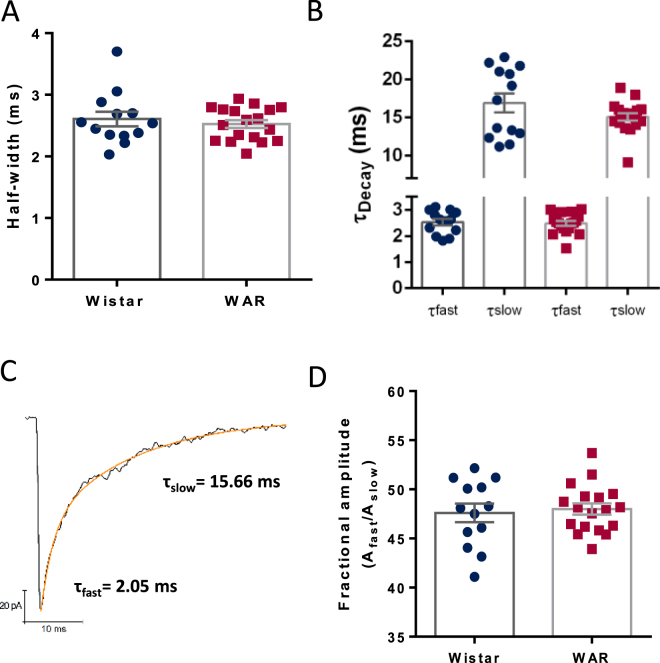
Kinetics of GABAergic mIPSCs. (**A**) Mean half-widths. (**B**) Mean fast and slow decay time constants obtained by the fittings of double exponentials. (**C**) Frequency distribution of fast and slow time constants. (**D**) The ratio between A_fast_ and A_slow_ shown as fast %. N = 13 cells from 7 Wistars and N = 18 from 7 WARs. N = 13 cells from 7 Wistars and N = 18 from 7 WARs.

Interestingly, amplitudes of the IPSCs were not affected by TTX. The amplitude distributions were similar in both groups (Fig. 4F,G. KS test, p = 0.9621) and the mean amplitudes of mIPSCs were not different between groups (Wistar = 74.46 ± 3.74 pA; WAR = 81.68 ± 4.4 pA; p = 0.32; Fig. 4H). Also, the CVs of the amplitudes were similar (Wistar = 59.7 ± 5.56%; WAR = 57.93 ± 4.83%; p = 0.81). We did not find differences in the half-widths (Wistar = 2.6 ± 0.12 ms; WAR = 2.5 ± 0.06 ms; p = 0.5; Fig. 5A) of the mIPSCs.

**Figure 6:**
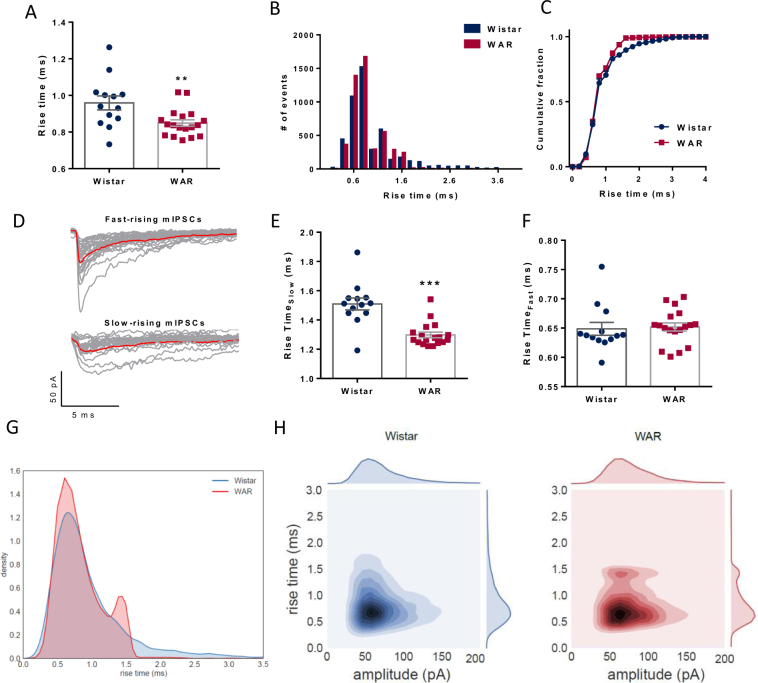
Rise times of quantal GABAergic mIPSCs. (**A**) Mean values for rise times. (**B**) Histogram showing distribution of events by rise times (Bins of 0.2 ms). Note the shape of two Gaussian distributions. (**C**) Frequency distribution of rise times. (**D**) Representative traces of mIPSCs divided in two groups; fast and slow rise time events. (**E**) Mean rise times for the group of fast rise times. (**F**) Mean rise times for the group of slow rise times. (**G**) KDE of the rise time distributions from the different groups where the Wistar KDE is plotted in blue and the WAR in red. (**H**) Bivariate KDE applied to rise time and amplitude distributions where darker colors represent higher densities. Each of the variables have its univariate KDE attached to the plot. **P < 0.01, ***P < 0.001.

The decay phase of the mIPSCs could be well fitted with double exponentials and characterized by a fast and a slow time constant both similar between groups (τ_fast_: Wistar = 2.53 ± 0.1 ms; WAR = 2.48 ± 0.09 ms; p = 0.7 and τ_slow_: Wistar = 16.9 ± 1.2 ms; WAR = 15.08 ± 0.48 ms; P = 0.13; Fig. 5B–D). Interestingly, we observed significant differences in the mean rise times (Wistar = 0.95 ± 0.04 ms; WAR = 0.84 ± 0.017 ms; p = 0.0078; Fig. 6A), indicating that mIPSCs in WAR cells were faster. In fact, the distribution of the rise times of both group of cells exhibited two peaks: a large peak at small values and a smaller peak for bigger values, indicating the existence of fast-rising mIPSCs (<1 ms) and slow-rising mIPSCs (>1 ms) (Fig. 6B–D). These distributions were statistically different between groups (KS test, p = 0.0027). We thus, separated the rise times in two groups (cutoff = 1 ms) and tested if the two populations of mIPSCs presented a similar pattern. We found that slow mIPSCs in cells from WARs had shorter rise times than from Wistars (Wistar = 1.5 ± 0.04 ms; WAR = 1.29 ± 0.019 ms; p < 0.0001, Fig. 6E), whereas both groups had similar fast mIPSCs (Wistar = 0.648 ± 0.01 ms; WAR = 0.65 ± 0.006 ms; P = 0.79, Fig. 6F). The fast and slow mIPSCs were similarly distributed among both strains, being 70.8% fast and 29.2% slow in cells from WARs and 65.4% fast and 34.6% slow in cells from Wistars. This result points to a particular change in the kinetics of mIPSPs. Whereas fast rise times are kept, longer rise times are altered. We propose that one subpopulation is kept (the fast), one subpopulation is lost (the slower) and one subpopulation is enhanced (peak close to 1.4 ms) (Fig. 6G). Markedly, if we combine the statistics from both groups (Fig. 6) the behavior is preserved. Amplitude is the same in both Wistar and WAR but we can still observe a second subgroup only in the WAR’s rise times as a darker region in the joint plot (Fig. 6H). Finally, no correlations were observed between rise times and amplitudes or half-widths (data not shown).

## Discussion

Although acute audiogenic seizures involve mainly the activation of primary brainstem auditory areas, chronic acoustic stimuli or audiogenic kindling often lead to the spread of epileptic activity to limbic areas, such as cortex, hippocampus and amygdala, resulting in a change in behavior and electrical activity^5–8,15,16^. At some point of audiogenic kindling, brainstem seizures and limbic seizures start to co-exist and this phenomenon can be easily observed with all audiogenic strains^4,16^. Interestingly, although, a few non-susceptible animals sometimes exhibit low score brainstem seizures, limbic seizures are rare, no matter how many stimulations the animals experience^17^, which leads to the hypothesis that in the audiogenic strains, the auditory-limbic pathways are altered. In fact, previous findings indicated that in the WAR strain, auditory-limbic circuitry is facilitated, even in the absence of seizures^18^. In this respect, audiogenic kindling, firstly described by Marescaux *et al*.^6^ and its electrophysiological counterparts subsequently reproduced in Genetically Epilepsy-Prone Rats (GEPRs) by Naritoku *et al*.^7^ and in WARs by Dutra Moraes *et al*.^8^ could be considered as a model of limbic recruitment or TLE^4,8^, the most common epilepsy syndrome in human adults^19^, in which a massive activation of limbic structures can be observed during seizures.

We have previously reported that, endogenously, dorsal hippocampi of WARs exhibited delayed LTP in Schaffer-CA1 synapses, which was accompanied by spatial memory impairments in the Morris Water Maze, suggestive of strain specific changes in hippocampal circuits^10^. In the current study, we investigated intrinsic and synaptic properties of CA1 pyramidal cells of WARs to verify the differences in the excitation/inhibition balance in the hippocampus of these animals that could underlie limbic recruitment. We found that CA1 pyramidal neurons have similar electrophysiological properties in WAR and Wistar control strains, except for a more hyperpolarized resting membrane potential in neurons from WARs.

To begin with, since it is well established that glutamatergic neurotransmission mediated by either AMPA/KA or NMDA receptors play an important role in seizure susceptibility in many animal models^2^, we first investigated possible alterations in excitatory synapses in the CA1 field of WAR. Our results show no differences in the excitatory glutamatergic AMPA/kainate and NMDA currents in both Wistar and WAR strains, suggesting that the fast-excitatory synapses in the CA1 are similar and could not be the substrate for the seizure susceptibility.

Similar to other cortical structures, most hippocampal cells are excitatory, and in CA1, only 11% of cells are GABAergic interneurons^20^. Despite that, inhibitory interneurons control the activity of principal cells and the loss of inhibition targeting excitatory neurons in the CA1, might have a profound impact not only in the hippocampus, but in areas that receive its connections^20^. In our study, we observed a decrease in spontaneous GABAergic currents whether the network was active or not. Since the spontaneous GABAergic inhibition strongly regulates pyramidal neuron firing^21^, the observed reduction in the frequency of sIPSCs in WARs could potentially impact the excitatory-inhibitory balance in these neurons, and potentiate their firing, when the Schaffer pathway is stimulated. In that context, a decreased GABAergic inhibition could favor the susceptibility to limbic seizures, as observed in audiogenically kindled WARs^4,8,16,22^. In fact, the impairment in the excitation/inhibition balance is thought as a possible trigger for seizures in many animal models of epilepsy and in clinical disorders^2^.

A decrease in the frequency of GABAergic currents could be consequence to a reduction in the number of interneurons or the number of synapses^23,24^. Many models of acquired and genetic epilepsy report alterations in GABAergic neurotransmission in the hippocampus. Knopp *et al*.^24^ observed a decrease in the frequency of mIPSCs in the subiculum of rats two weeks after *Status Epilepticus* (SE) induced by pilocarpine (PILO). These authors observed a marked decrease in the number of fast spiking parvalbumin^+^ interneurons in the CA1 pyramidal layer and subiculum. Although we did not see a decrease in mIPSC frequency, our data showed that mIPSCs in WAR were separated by longer inter-event intervals, what could also reflect a change in the number of active synapses or release probability. According to Wierenga and Wadman^25^, hippocampal kindling induces a reduction in the frequency of mIPSCs in CA1 pyramidal cells. It is important to remember that in the PILO model, intense death of interneurons is always reported^26^, in contrast to what happens in hippocampal kindling^27^, where a modest cell loss can be observed. In both models, a re-organization of synaptic inputs take place in the hippocampus preceding the appearance of seizures and continuing as seizures occur. In our model, WAR animals do not present Fluorojade+ (Schmued *et al*., 1997) neurodegenerated cell counts or mossy fiber sprouting, such as the one presented after, for example, PILO-induced SE (Leite *et al*., 2001) different from Wistar controls after audiogenic kindling in the hippocampus^22^ with a subtle but significant reduction of cell counts in perirhinal cortex and amygdala^10^ after audiogenic kindling. However, in order to have a precise evaluation of the impact of these alterations in WARs limbic (hippocampal-amygdala, perirhinal) networks, an estimation of the number of GABAergic interneurons and their subtypes compared to Wistar parental strain is eagerly needed. We found a fast-rising and a slow-rising mIPSCs, as previously described in animals exposed to hippocampal kindling^25^. However, in contrast to those findings, we observed a change in mIPSC kinetics, with a decrease in the mean rise times of mIPSCs. We found that “slow” rise time population of WARs is faster, which could be explained by changes in GABA_A_ receptor subunit composition. Furthermore, this effect could also be a result of the presence of more proximal synapses (to be proven by microscopy studies) in WARs, resulting in decreased dendritic filtering, even in the absence of correlation between rise time amplitude or decay time constants of recorded mIPSCs^28–30^. In fact, Labrakakis *et al*.^31^ showed that events with slower rise times originating from dendritic synapses are not always smaller in amplitude, as if they were a result of electrotonic filtering. We thus believe that this change in mIPSC kinetics may result from a combination of subunit composition of the GABAA receptors and synapse location. Llano *et al*.^28^ found also in Purkinje cells two populations of mIPSCs regarding their rise times. A fast population that originate from synapses located on the soma or proximal dendrites and slow rise times population that originate at distal dendrites. Therefore, the finding that WARs mIPSCs rise times are faster would indicate that most events come from somatic origin and consequently, neurons from these animals have less synapses coming from dendrites, which might impair the ability for dendritic inhibitory inputs to efficiently hamper the excitatory signals, contributing to the susceptibility to seizures. We are currently investigating if this is the case using electronic microscopy.

We finally report that WAR hippocampal pyramidal CA1 neurons are more hyperpolarized and fire less action potentials than Wistar hippocampal cells. Since the action potential threshold was not different in WARs, we consider that the decrease in firing is a consequence of lower resting membrane potential. In our interpretation, this alteration in resting membrane potential, could be a result of intrinsic homeostatic plasticity that counterbalances the deficient inhibition, protecting the cell from hyperexcitation. Indeed, it has been demonstrated that after chronic increases in network excitability, cellular changes will often reduce intrinsic excitability^32^.

We conclude that due to specific changes in fast GABAergic synapses, WAR, a genetically developed strain susceptible to epilepsy, can be a good model to understand how deficient GABAergic neurotransmission behaves when challenged, with potential impact in the control of excitability, such as the one associated to epilepsy. Although the animals studied in the present study are seizure naïve, we can speculate that if further audiogenic kindling is imposed to them, the presence of hyperexcitability would be facilitated and this is the aim of ongoing and future investigations. We therefore propose that the interaction of WAR genetic background with the chronic seizure experience, in this case, driven by high intensity sound stimulation, could mimic processes that underlie some types of human epileptic disorders.

## Methods

All experiments were performed according to rules for animal research from the National Council for Animal Experimentation Control in Brazil (CONCEA). All experiments in this study were approved by the Ethics Committee for Animal Use (CEUA) at the University of São Paulo in Ribeirão Preto, protocol # 015/2013.

### Animals

Wistar Audiogenic Rats (WARs) were inbred and raised at the Central Animal Housing Facility at the University of São Paulo as previously described^33^. For both strains, we used male rats (60–80 days, 5–8, per group), kept in Plexiglas cages (2–3 animals per cage), food and water ad libitum and 12-h dark/light cycle (lights on at 7:00 a. m.) and controlled temperature (22 °C). We used naïve animals to avoid seizure-related effects.

### Hippocampal Slices

Animals were anesthetized with isoflurane and quickly decapitated. Brains were carefully removed and placed in an ice-cold solution containing (mM): 87 NaCl, 2.5 KCl, 25 NaHCO_3_, 1.25 NaH_2_PO_4_, 75 Sucrose, 25 Glucose, 0.2 CaCl_2_, 7 MgCl_2_, bubbled with 95% O_2_ and 5% CO_2_. Brains were glued with cyanoacrylate glue to a support, placed inside the cutting chamber of a vibratome (1000 plus, Vibratome, USA) and cut in 200 µm transversal slices containing the dorsal hippocampus. The slices were placed in aCSF solution containing (mM): 125 NaCl, 2.5 KCl, 1.25 NaH_2_PO_4_, 26 NaHCO_3_, 10 Glucose, 2 CaCl_2_, 1 MgCl_2_ for one hour at 34–35 °C and thereafter for at least one hour at room temperature before recordings. Only sections 3.6 to 4.5-mm anterior-posterior with respect to bregma were used^34^.

### Whole Cell Patch Clamp Recordings

CA1 pyramidal neurons were visualized with an Olympus BX51WI Microscope (Olympus, Japan) with infrared differential interference contrast (IR-DIC). Neurons were chosen based on the morphology (pyramidal shape) and position in the pyramidal layer. We chose neurons located in the middle of the layer to avoid electrophysiological differences between superficial and deep pyramidal cells^35^.

Patch clamp recordings were performed using a Heka EPC10 (HEKA Elektronik, Germany) amplifier with 50 kHz sampling rate and low pass filtered at 3 kHz (Bessel). The slices were placed in the recording chamber filled with aCSF and controlled temperature at 34 °C (Scientifica, UK). To record intrinsic properties of neurons, we used an internal solution consisting of (mM): 138 K-gluconate, 8 KCl, 10 Hepes, 0.5 EGTA, 10 phosphocreatine, 4 Mg-ATP, 0.3 Na-GTP adjusted to pH 7.3 with KOH and ≈290 mOsm/kgH_2_O. To record glutamatergic excitatory post-synaptic currents (EPSCs) we stimulated Schaffer collaterals with a concentric bipolar microelectrode (FHC – Bowdoin, ME, USA), always placed in a visually-controlled distance from the recorded cell, connected to a SD9 Grass voltage stimulator (Natus Medical Incorporated, Warwick, RI, USA). To record AMPA and NMDA-EPSCs, we used an internal solution consisting mM): 130 CsCl, 10 Hepes, 5 EGTA, 5 phosphocreatine, 4 Mg-ATP, 0.5 Na-GTP, 10 TEA, 5 QX 314 adjusted to pH 7.3 with CsOH and ≈290 mOsm/kgH_2_O. We used the minimum voltage necessary to evoke the maximum current and recorded the EPSCs at holding potentials from −70 mV to +80 mV, increments of +30 mV. We applied DNQX to block AMPA/KA currents and isolate NMDA currents. AMPA/KA IVs were obtained by subtracting the currents before and after DNQX and the NMDA-AMPA ratio was obtained in the same cell by dividing the current evoked at +80 by the current evoked at −70 mV. We confirmed that currents were mediated by NMDA receptors using the NMDA antagonist, D-AP5 (10 µM). In some experiments, we recorded AMPA-mediated glutamatergic evoked currents with KGlu based internal solution. EPSCs recorded with KGlu and CsCl were not significantly different, so we decided to group them together. Glutamatergic currents were recorded in the presence of picrotoxin (20 µM). All evoked and spontaneous synaptic currents were blocked by a combination of PTX (20 µM), DNQX (10 µM) and D-AP5 (10 µM).

Spontaneous GABAergic currents were recorded in the presence of 6,7-dinitroquinoxaline-2,3-dione (DNQX) with an internal solution consisting of (mM): 145 KCl, 10 Hepes, 0.5 EGTA, 10 phosphocreatine, 4 Mg-ATP, 0.3 Na-GTP, adjusted to pH 7.3 with KOH and ≈290 mOsm/kgH_2_O. In order to test if we could improve the detection of dendritic synaptic currents we a recorded some cells (n = 6) with CsCl-based internal solution, which could improve space-clamp control in the dendrites by reducing leak potassium conductances and compared the IPScs with the ones recorded in KCl. We found that IPSCs recorded in CsCl had similar parameters than IPSCs recorded in KCl, except by a small increase in the decay time (Data not shown). We conclude that the CsCl internal solution does not improve the detection of distal IPSCs, as suggested by a previous work^36^.

Electrodes were fabricated from borosilicate glass (BF150-86-10, Sutter Instruments, Novato CA) with tip resistances of 3–5 MΩ. Series resistance (<20 MΩ) was compensated in 60%. Any neuron with series resistance increased over 20% during experiments, as well as resting membrane potential higher than −60 mV, was discarded. Voltages were corrected off-line for a liquid junction potential for each internal solution calculated with Clampex software (Molecular Devices).

### Drugs

The following drugs were used: picrotoxin (Sigma, 20 and 100 µM), 6,7-dinitroquinoxaline-2,3-dione (DNQX - Sigma, 10 µM), D-(-)-2-Amino-5-phosphonopentanoic acid (D-AP5 - Tocris, 10 µM) and tetrodotoxin (TTX - Cayman Chemical, 0.5 µM). DNQX was dissolved in DMSO and then added to bath from fresh stock solutions. The final concentration of DMSO in the experiments was 0.1% and since we did not find differences between DMSO and aCSF, we used only aCSF as control vehicle. All salts were of reagent grade.

### Data Analysis

Data was analyzed using Mini Analysis (Synaptosoft 6.0.3, Fort Lee, NJ, USA) and custom written routines in IgorPro (Wavemetrics, Portland, OR, USA) and Matlab (MathWorks, Natick, MA, USA). Voltage-current relations (VI) were built measuring the steady-state voltage change in response to hyperpolarizations and we measured membrane input resistance, membrane time constants and the depolarization sag. Step depolarizations were used to produce the firing frequency-intensity curves (FI). We used the first action potential fired to obtain from phase-plane plots (dV/V): threshold, rate of rise (ROR), half-width and fast afterhyperpolarization (AHP).

The peaks of the EPSCs were used to build IV relationships, to calculate the reversal potential and to estimate the chord conductances. We measured rise times from baseline to peak and decay times from peak to baseline.

GABAergic currents were recorded at −70 mV. Spontaneous inhibitory postsynaptic currents (sIPSCs) were recorded for 10 minutes. We next applied TTX to block action potentials and record miniature IPSCs (mIPSCs). IPSCs and mIPSCs were selected manually, and only currents with good signal to noise ration were chosen. For each recording file, we tested the RMS noise and compared among groups. Since the electrical noise was similar among recordings, we chose not to use any type of offline filter to avoid distortions in the kinetics of events. Histograms for interevent intervals, amplitudes and rise times were built with same fixed bins for different groups of cells and cumulative frequency distributions were tested for significance with Kolmogorov-Smirnov (KS) test. Analysis of decay kinetics for inhibitory currents was performed by Mini Analysis group analysis with individual currents fitted with double exponential functions. Fast and slow time constants were presented as average and compared between groups. Further analysis of the mIPSC amplitude and rise time distributions was made using kernel density estimation (KDE). KDE was estimated using Seaborn library for the Python programming language (Python Software Foundation, https://www.python.org/).

Data from other experiments are reported as mean ± SEM and were tested with unpaired t-test, considering p < 0.05. Also, we used two-way ANOVA to test for significance in FI curves and train stimulation of Schaffer collaterals.

### Ethical Publication Statement

We confirm that we have read the Journals position on issues involved in ethical publication and affirm that this report is consistent with those guidelines.
